# Randomized controlled study comparing the use of diphencyprone and anthralin in the treatment of extensive chronic alopecia areata^☆^^☆☆^

**DOI:** 10.1016/j.abd.2020.06.018

**Published:** 2021-03-16

**Authors:** Vanessa Barreto Rocha, Priscila Kakizaki, Aline Donati, Carla Jorge Machado, Mario Cezar Pires, Leticia Arsie Contin

**Affiliations:** a Instituto de Assistência Médica do Servidor Público Estadual, São Paulo, SP, Brazil; b Hospital do Servidor Municipal de São Paulo, São Paulo, SP, Brazil; c Departamento de Saúde Pública, Faculdade de Medicina, Universidade Federal de Minas Gerais, Minas Gerais, MG, Brazil

Dear Editor,

Alopecia Areata (AA) does not have a well-established etiopathogenesis and its treatment, especially in the chronic form, remains a challenge.^1^

Its clinical spectrum varies from a single plaque to total alopecia (total AA [TAA]) or universal alopecia (UAA) when it affects all body hair. The percentage of hair loss is one of the unquestionable prognostic factors and can be measured using the SALT (Severity of Alopecia Tool) score.^2^, ^3^ UAA and TAA are the types that are most refractory to treatment, and their spontaneous hair regrowth rate is less than 10%.^4^

Diphencyprone (DPCP) is one of the most studied treatments for extensive AA, applied weekly, usually by the doctor.^5^ Anthralin is an old drug, which is used at home and has a lower cost, with few studies carried out on its use in AA.^6^, ^7^, ^8^, ^9^, ^10^ Both medications act by inducing eczema over the alopecic area, with DPCP causing allergic contact dermatitis (CD) and anthralin, irritative CD.

Therefore, it can be assumed that the results of the two drugs are similar; however, there are no studies comparing the effectiveness of anthralin and DPCP in the treatment of AA.

The aim of this study was to compare the efficacy, tolerability, and safety of the two drugs in the treatment of chronic extensive AA (more than one year of duration).

A randomized, controlled clinical trial was performed at the Dermatology Trichology Outpatient Clinic at the Hospital do Servidor Público Municipal de São Paulo - Brazil. After approval by the Ethics Committee (CAAE nº 60888516.1.0000.5442) and signature of the informed consent form, 24 patients who had AA for more than one year, SALT score ≥50, and more than 30 days without treatment, were included and randomly allocated for receiving DPCP (n = 13) (with gradual dose increase until mild eczema was attained) or 2% anthralin in petroleum jelly for 30 min (n = 11). There was an intra-patient control through the initial application only in the right hemicranium. The percentage of hair regrowth was calculated through photos, by a single examiner who was blinded to the treatment group. Because of the characteristics of the medications, it was not possible for the professional applying the medication or the patient to be blinded to the substance used.

The evaluations were carried out at three months, or when there was initial hair regrowth, then at six months. The proposed treatment time was 24 weeks.

The study was funded by the Dermatology Support Fund of the Brazilian Society of Dermatology (FUNADERM).

Out of the 24 patients included, three had total AA (12.5%), 13 UAA (54.2%) and eight had extensive multifocal AA (33.3%). Clinical, demographic, and hair regrowth data are shown in Table 1.

**Table 1:** Clinical, epidemiological data and comparison regarding hair regrowth in the DPCP and anthralin groups.

Variable	DPCP, n (%)	Anthralin, n (%)	p
Sex	11F / 2M	10F / 1M	>0.999
Age, mean (DP)	36.8 (10.7)	34.1 (7.0)	0.474
Clinical type			
Multifocal	5	3	0.093
TAA	2	1	0.642
UAA	6	7	0.392
Age at the 1^st^ episode, mean (SD)	23.7 (14.8)	17.8 (11.3)	0.293
**Episode duration in months**			
Median (IQR)	36 (108)	24 (102)	0.675
Minimum/Maximum	8/192	8/240	
Atopy	4 (30.8)	5 (45.5)	0.675
Ungual alterations	3 (23.1)	3 (27.3)	>0.999
Anti-H1 use	2 (15.4)	1 (9.1)	>0.999
**Previous treatments**			
Oral corticoid	8 (61.5)	8 (72.7)	0.562
Corticoid infiltration	6 (56.2)	8 (72.7)	0.240
Topical corticoid	7 (43.8)	4 (36.4)	0.444
Minoxidil	5 (38.5)	7 (63.6)	0.414
IV Corticoid	1 (7.7)	0 (0.0)	>0.999
Anthralin	1 (7.7)	2 (18.2)	0.576
DPCP	2 (15.4)	3 (27.3)	0.630
Methotrexate	1 (7.7)	3 (27.3)	0.300
Chloroquine	0 (0.0)	1 (9.1)	0.458
**Results**			
Initial SALT score			
Median (IQR)	98 (28.5)	10 (7.2)	0.178
Minimum / Maximum	60.1 / 100	93.4 / 100	
6-month SALT score			
Median (IQR)	89.9 (29.0)	99.9 (0.72)	0.242
Minimum / Maximum	60.9 / 100	75.1 / 100	

SDP, Standard Deviation; IQR, Interquartile Range; Anti-H1, Anti-Histaminic drugs; IV, Intravenous.

To compare the means, Student’s *t* test was used for independent samples; the non-parametric Wilcoxon and Mann-Whitney test was used to compare the medians. To compare proportions, Fisher's exact test was performed. All analyses were performed considering Intention-to-Treat (ITT) and per protocol. The level of significance was set at 5% (p < 0.05; two-tailed test).

As for hair regrowth, the improvement was small and similar in the DPCP and anthralin groups (Figure 1, Figure 2). The group treated with DPCP showed some hair regrowth in 38.5% of the cases (5/13) and the anthralin group in 18.2% (2/11), a difference without statistical significance (p > 0.05). No patient had more than 75% of hair regrowth in this study. The side effects were similar in both groups (p = 0.121 for 3 months and p = 0.617 for 6 months).

**Figure 1:**
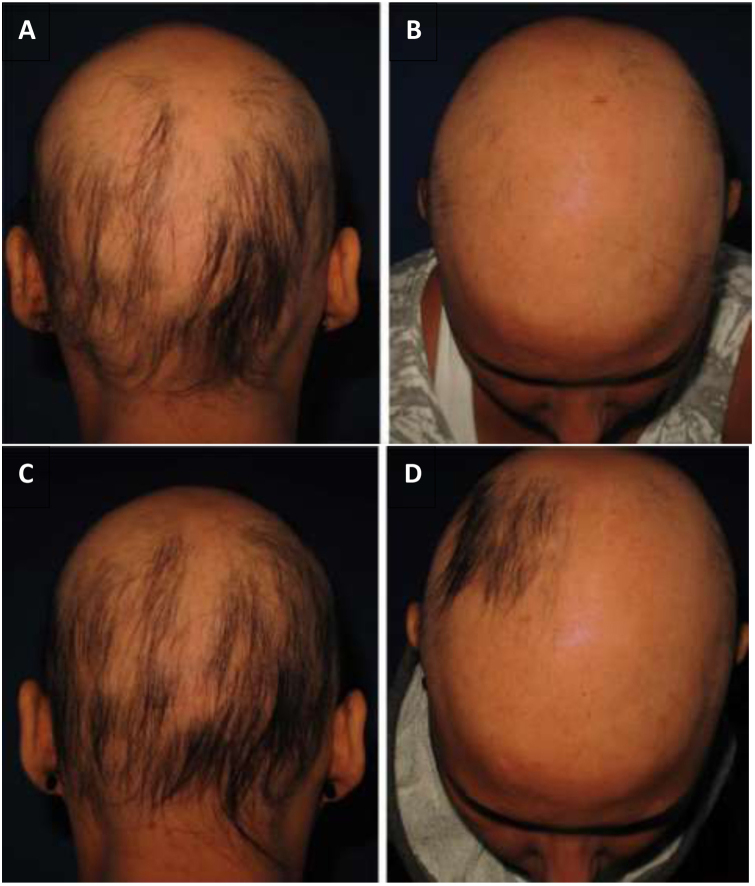
(A and B) DPCP – initial appointment; (C and D) DPCP – 17 applications. The patient was treated with 17 applications of DPCP and presented hair regrowth. (A and B) Initial assessment; (C and D) after 17 applications of DPCP.

**Figure 2:**
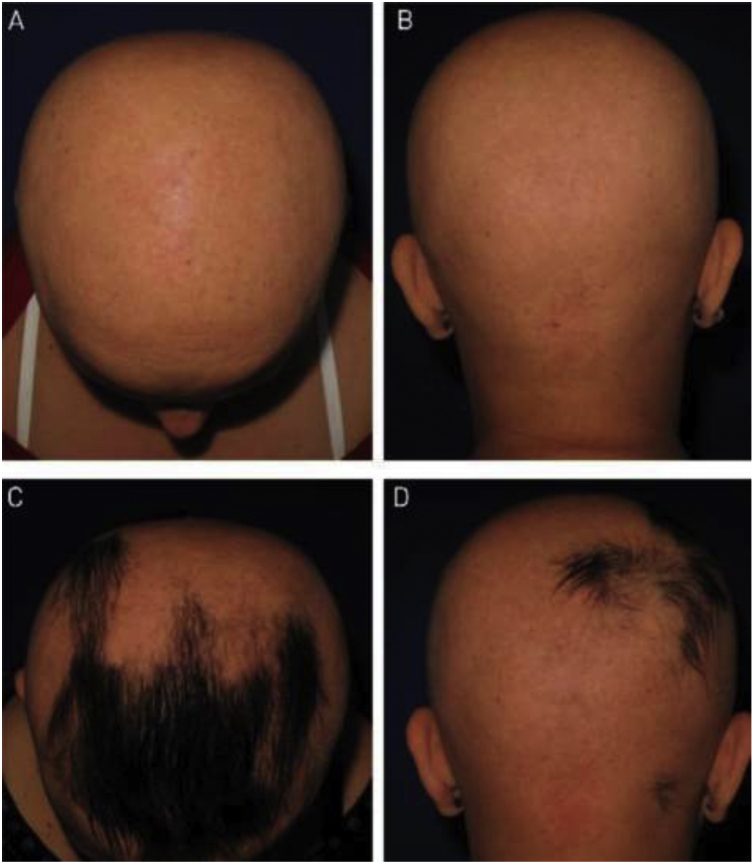
Anthralin: (A and B) Initial pictures; (C and D) after 24 weeks of treatment. Patient was treated with Anthralin for 24 weeks. (A and B) Initial assessment; (C and D) after 24 weeks of treatment.

A recent review found 31 studies using DPCP to treat AA, with 1,638 treated patients, but none of them were randomized or placebo-controlled studies. There was no hair regrowth in 30.7% (22.9% –39.6%) for patients with TAA and UAA, similar to that found in our group.^5^ In this review, the incidence of severe eczema was 30.8%, followed by lymphadenomegaly (25.8%), generalized eczema (15.8%), hyperchromia (12.7%), and influenza-like symptoms (11.1 %).^5^

In our case series with DPCP, only one patient had moderate eczema and two had urticaria.

Three patients were lost of follow up in the DPCP group, due mostly to problems with coming to the clinic weekly. Of the 13 patients treated with DPCP, seven finished the 24 weeks of application, with an average of 18.4 sessions.

Anthralin is used both in children and adults with AA but there have been few studies on its use, which were mainly conducted in adults.^6^, ^7^, ^8^, ^9^, ^10^ Özdemir and Balevi (2017) treated 30 children (6.7% with UAA and 3.3% with TAA) with 1% anthralin in one hemicranium for 20–60 min daily. The onset of hair regrowth was observed on average after three months of treatment, and the maximum response after nine months. Eleven patients (36.6%) attained a partial response. Nine patients (30%) who did not respond to treatment after nine months were excluded from further statistical analysis. Patients who did not respond had a higher average SALT score than those who responded completely or partially.^8^

As for anthralin, in our study, two patients (18.2%) showed some improvement in the SALT score. The 11 patients had erythema and pruritus, which were generally mild, and only one had moderate eczema. Most had scalp hyperpigmentation, an expected fact and a sign of adherence to treatment, which reversed a few weeks after anthralin discontinuation. In this group, two patients were lost, one due to disease activity, with significant worsening of alopecia, and the other due to personal problems.

As study limitations, this is a small sample, justified by difficulties in patient selection and the project budget. The literature has been working with small samples due to the aforementioned difficulties.^5^ The time of follow-up was short, only six months, often due to the participants' impatience and anxiety, who requested a change of treatment after this period.

The extent of alopecia in the groups of this study may justify the low response observed. The studied population is the one with the worst prognosis and without concomitant treatment, unlike most studies in the literature.

Although a longer time of follow-up was not achieved, this is the first prospective study comparing the effectiveness of DPCP and anthralin. In conclusion, there was no statistical difference between the responses to both treatments.

## Financial support

FUNADERM - Fundo de Apoio à Dermatologia.

## Authors’ contributions

Vanessa Barreto Rocha: Statistical analysis; design and planning of the study; drafting and editing of the manuscript; collection, analysis, and interpretation of data; effective participation in research orientation; intellectual participation in the propaedeutic and/or therapeutic conduct of studied cases; critical review of the literature.

Priscila Kakizaki: Approval of the final version of the manuscript; design and planning of the study; data collection; intellectual participation in the propaedeutic and/or therapeutic conduct of studied cases; critical review of the manuscript.

Aline Donati: Statistical analysis; approval of the final version of the manuscript; design and planning of the study; analysis and interpretation of data; effective participation in research orientation; intellectual participation in the propaedeutic and/or therapeutic conduct of studied cases; critical review of the literature; critical review of the manuscript.

Carla Jorge Machado: Statistical analysis; approval of the final version of the manuscript; design and planning of the study; analysis and interpretation of data; effective participation in research orientation; critical review of the manuscript.

Mario Cezar Pires: Approval of the final version of the manuscript; effective participation in research orientation; intellectual participation in the propaedeutic and/or therapeutic conduct of studied cases; critical review of the manuscript.

Leticia Arsie Contin: Approval of the final version of the manuscript; design and planning of the study; drafting and editing of the manuscript; collection, analysis, and interpretation of data; effective participation in research orientation; critical review of the manuscript.

## Conflicts of interest

None declared.
